# UNDERSTANDING THE IMPACT OF OBESITY THROUGH THE LIFECOURSE ON LATER-LIFE FUNCTIONING AND DEPENDENCY

**DOI:** 10.1093/geroni/igz038.189

**Published:** 2019-11-08

**Authors:** Carol Jagger

**Affiliations:** Newcastle University, Newcastle upon Tyne, United Kingdom

## Abstract

Populations worldwide are seeing rising levels of obesity and its health consequences, particularly diabetes. Levels of childhood obesity are particularly high with concerns of how this will affect individuals’ health and functioning in mid and later life. Such research questions are difficult to answer as ideally they require longitudinal studies of cohorts from birth or childhood through to later life, with consistent measures of obesity and functioning throughout. The first two presentations in this session use the unique UK birth cohorts, the 1946 National Survey of Health and Development (1946-NSHD) and the 1958 National Child Development Study (1958-NCDS) with a focus on poor physical functioning (PF, i.e. the ability to perform physical tasks of daily living) in later life. Poor PF was defined as the lowest (gender and cohort-specific) 10% on the Short-form 36 subscale at 60-64y (1946-NSHD) and 50y (1958-NCDS). The presentations explore (i) how the timing of onset and duration of obesity, from childhood through to mid-life, affects later life PF, and (ii) whether the relationship between obesity and PF is mediated by physical inactivity. In the final presentation we utilise a new dynamic micro-simulation model, the Population Ageing and Care Simulation (PACSim) which simulates the ageing of a base population of individuals aged 35 years and over from three longitudinal studies (Understanding Society, the English Longitudinal Survey of Ageing, and the Cognitive Function and Ageing Study II) to examine the extent to which reducing obesity in mid life could potentially reduce later dependency and care needs.

